# Veteran Experiences and Satisfaction With Veterans Affairs Call Centers’ Tele-Triage and Virtual Urgent Care Appointments: Qualitative Evaluation

**DOI:** 10.2196/80656

**Published:** 2025-11-11

**Authors:** Caroline Gray, Allison Engstrom Buggaveeti, Tracy Urech, Anita Vashi

**Affiliations:** 1 Center for Innovation to Implementation (Ci2i), VA Palo Alto Health Care System, 795 Willow Road, Menlo Park, CA, 94025, United States, 1 6502486147

**Keywords:** telemedicine, triage, virtual health, veteran, health services, patient satisfaction, health care delivery, qualitative research

## Abstract

**Background:**

Virtual health care models that incorporate registered nurse triage with rapid access to same-day virtual visits with clinicians represent a growing innovation in health care delivery. While traditional telephone advice lines focus primarily on registered nurse–led triage and care navigation, systems such as the Department of Veterans Affairs (VA) are beginning to embed physicians and advanced practice providers directly into these platforms. This hybrid model has the potential to enhance clinical responsiveness, reduce unnecessary emergency department and urgent care visits, and increase patient satisfaction by providing timely care from home.

**Objective:**

This study aimed to explore Veterans’ experiences and perceptions of the VA’s integrated virtual triage and urgent care model, specifically through the VA Health Connect platform. We sought to understand how Veterans learned about and interacted with these services and to gather their insights on aspects to preserve or improve.

**Methods:**

We conducted in-depth qualitative interviews with 24 Veterans from various geographical regions served by 6 VA health care systems. Interviews were carried out between June 18 and August 8, 2024. Data were analyzed using a qualitative descriptive approach with constant comparison to identify emergent themes and representative quotes.

**Results:**

Participants reported high satisfaction with VA Health Connect’s nurse triage and virtual clinical visit services. Key benefits included timeliness of care, personal time savings, efficient service organization, and positive interactions with nurses and providers. Veterans appreciated the convenience of resolving health issues quickly and remotely, often citing significant travel burdens avoided. They also highlighted the knowledgeable and personalized clinical advice received. However, several areas for improvement were identified. Some Veterans expressed frustration about being routed to nurse triage instead of directly scheduling with their primary care providers. Moreover, many were initially unaware of the full range of services available through VA Health Connect and suggested enhanced outreach and communication strategies.

**Conclusions:**

Veterans are highly satisfied with the VA Health Connect model, valuing its timeliness, convenience, and the professionalism of clinical staff. Effective promotion and clear communication about the capabilities and limitations of the service could further enhance user experience and uptake. As this integrated care model continues to evolve, its success will depend on effectively integrating virtual services into routine care and ensuring Veterans are well-informed and confident in using these resources.

## Introduction

Virtual care models that integrate registered nurse (RN) triage with rapid access to same-day virtual visits with clinicians are a relatively new and growing innovation in health care delivery. Traditionally, telephone advice lines have focused on RN-led triage and care navigation. However, health systems, including the Department of Veterans Affairs (VA), are beginning to embed physicians and advanced practice providers directly into these platforms to evaluate, treat, and follow up with patients in real time. This hybrid model offers the potential to improve clinical responsiveness, reduce unnecessary emergency department and urgent care use, and enhance patient satisfaction by delivering timely care from the convenience of home. Early evidence suggests that outcomes for patients treated remotely for acute conditions are comparable, and in some cases superior, to in-person care [1,2]. At the same time, these services may reduce out-of-pocket costs, travel time, and long wait times, which are common sources of dissatisfaction in traditional acute care settings [3,4]. Despite growing adoption, little is known about how patients experience and perceive this evolving model of care.

**Figure 1.**
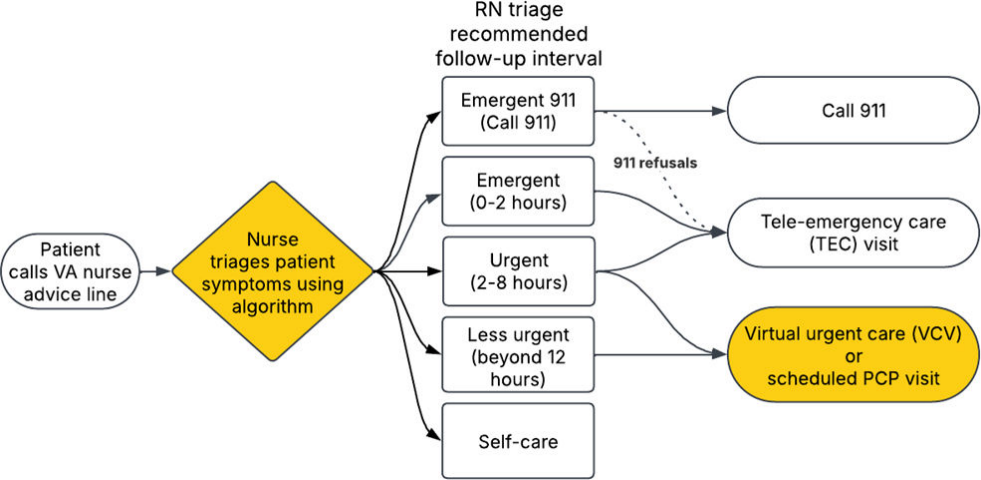
Veterans Affairs Health Connect remote triage and urgent care workflow. PCP: primary care provider; RN: registered nurse; TEC: tele-emergency care; VA: Veterans Affairs; VCV: virtual clinical visit.

The VA has emerged as a national leader in implementing integrated virtual triage and urgent care services across its health care system. Central to this effort is VA Health Connect, a nationwide program of Clinical Contact Centers, or more colloquially referred to simply as call centers, that delivers telephone and video-based services to Veterans. VA Health Connect functions as a system-wide program designed to standardize access to timely, patient-centered care. While VA Health Connect is a national program, each call clinical contact center serves designated VA medical centers. VA Health Connect supports and offers Veterans four core services: (1) scheduling and administrative support, (2) pharmacy and medication refill requests, (3) nurse triage and symptom management, and (4) virtual clinical visits (VCV) with urgent care providers. Nurse triage and VCV services are closely coordinated. Veterans who call with health concerns are first routed to an RN, who conducts a structured assessment using standardized protocols and integrated decision-support tools. Based on clinical acuity, provider availability, and patient preference, the nurse may refer the Veteran for a same-day virtual visit with a provider (Figure 1).

To expand clinical capacity and improve timely access to urgent care, the VA launched its VCV service in 2020 as a regular component of VA Health Connect. This addition marked a shift from traditional nurse–only advice lines by embedding same-day access to treating clinicians, typically nurse practitioners with urgent or emergency care expertise, directly into the triage workflow. VCV providers perform evaluations, initiate treatment, prescribe medications, order diagnostics, and coordinate follow-up care as needed. By resolving acute issues remotely, the service reduces travel burdens, especially for rural or mobility-limited Veterans, and supports more efficient use of system resources. Evaluations of VCV and related programs, such as the VA’s Tele-Emergency Care initiative, have demonstrated safety, effectiveness, and reductions in downstream emergency department use [5,6].

While system-level outcomes have been assessed, less is known about how patients perceive and experience these services. Traditional satisfaction surveys suggest high approval ratings but may not capture the full range of patient experiences, expectations, or barriers. As the VA and other health care systems expand virtual care, understanding the patient perspective is essential to improving design, communication, and uptake. Gaining a deeper understanding of the patient journey is critical to advancing patient-centered care [7], which is strongly associated with better clinical outcomes and more appropriate care use.

To address this gap, we conducted a qualitative study of Veterans who used VA Health Connect’s nurse triage and virtual urgent care services. We examined how Veterans became aware of these services, their interactions with clinicians and staff, and their perspectives on which aspects of the services should be preserved or improved.

## Methods

### Participants and Study Design

As part of a broader case-based qualitative evaluation of the modernization of VA’s call centers, we conducted in-depth interviews with 24 Veterans from geographically diverse areas served by 6 VA health care systems. Interviews were conducted between June 18 and August 8, 2024. Participants were identified through V-Signals Survey responses for having had both a nurse triage and a virtual visit with a provider. V-Signals is a routinely administered “pulse” survey designed to gather rapid, real-time feedback from Veterans about their experiences with VA services [8]. The survey is regularly sent to Veterans who have received care through VA Health Connect, including nurse triage line interactions and visits with virtual providers. It includes a question asking whether respondents would be willing to discuss their experiences further with someone from VA.

To facilitate timely recruitment and ensure accurate recall of experiences, we limited outreach to Veterans who had recently completed a V-Signals survey and indicated willingness to be contacted. Veterans were emailed from a dedicated VA Health Connect Evaluation email address created specifically for this project. Of the 168 Veterans contacted, 44 responded. Out of these 44 Veterans, 16 expressed initial interest but did not follow through with scheduling. Overall, 4 Veterans declined participation. Ultimately, 24 Veterans completed an interview. The mean time between the Veteran’s V-Signals survey and the interview was 20 (SD 4) days. Participant characteristics are summarized in Table 1.

**Table 1.** Veteran characteristics.

Characteristics	Participants (N=24)
Age (years), mean (SD)	66.3 (12.4)
Sex, n (%)	
Female	5 (20.8)
Ethnicity, n (%)	
Hispanic	2 (8.3)
Non-Hispanic	22 (91.7)
Race, n (%)	
American Indian or Alaska Native	1 (4.2)
Black or African American	2 (8.3)
White	20 (83.3)
Missing	1 (4.2)
Resides in a rural location, n (%)	8 (33.3)
Geographic region, n (%)	
Northeast	8 (33.3)
South	12 (50)
West	4 (16.7)

### Data Collection and Analysis

A trained qualitative social scientist conducted the interviews using Microsoft Teams. The evaluation team developed and, based on input from operational partners, modified an interview guide prior to data collection. The interview guide was revisited throughout the data collection process to ensure questions were capturing rich and useful information. Interviews explored Veterans’ overall experiences with the nurse triage and VCV services, areas for improvement, understanding of VA Health Connect and its offerings, and suggestions for increasing awareness of these services among Veterans. Operational partners from VA’s Office of Integrated Care reviewed the interview guide and provided additional questions for inquiry. The final version of the guide is included in Multimedia Appendix 1.

With participant consent, interviews were audio-recorded and transcribed by a professional transcription service. The interviewer also drafted summaries after each session to support real-time generative analysis and enable debriefings with the broader evaluation team. These debriefings informed ongoing adjustments to the interview guide, including the addition of follow-up questions and probes—an approach consistent with iterative qualitative data collection and analysis. Interview content was also assessed throughout the data collection phase to ensure sufficiency for the identification of themes and subthemes. Sufficiency is often used as an alternative to saturation because of the latter’s limited applicability to more applied research inquiry [9].

Interview transcripts were reviewed using a qualitative descriptive approach using constant comparison [10] to further reduce and synthesize data [11]. First, the evaluation team inductively reviewed 5 transcripts alongside the interview guide to identify emergent codes as well as deductive codes derived from the interview questions. Transcripts were fully coded by the lead qualitative analyst on the team and the first author (CG). A secondary coder (AE) reviewed additional transcripts to ensure coding reliability. A pragmatic coding scheme was used, limiting the need for coding disagreement resolution to take place. Transcripts were uploaded into ATLAS.ti (ATLAS.ti Scientific Software Development GmbH), a software that facilitates qualitative analysis, and were coded according to a collaboratively developed codebook. Once coding was complete, team members reviewed and grouped codes into broader categories. During this process, we identified themes by looking for repetition and emphasis of specific points. Representative quotes were then selected to illustrate each of the major themes presented in the Results section.

### Ethical Considerations

This quality improvement project was reviewed and designated as nonresearch by Stanford University’s Institutional Review Board. The Institutional Review Board has determined that this project does not meet the definition of research as defined in 45 Code of Federal Regulations (CFR) 46.102(d), nor the definition of clinical investigation as defined in 21 CFR 50.3(c). Despite being categorized as nonresearch, oral informed consent was obtained from all participants prior to their involvement in the study. Permission was also granted to audio-record the interviews. All audio recordings were transcribed, and the transcripts were deidentified to ensure the anonymity of participants. This deidentification step was taken to protect the privacy and confidentiality of the participants. Participants were compensated with US $50 for their involvement in this project.

## Results

### Benefits of VA Health Connect

Overall, interview participants were extremely satisfied with their experiences using the nurse triage and VCV services of VA Health Connect. When asked what they valued most, Veterans emphasized several key aspects: (1) the timeliness of care and time savings, (2) helpful clinical advice and recommendations, (3) organizational efficiency, and (4) positive interactions with nurses and providers.

#### Timeliness, Efficiency, and Time Savings

Timeliness, or specifically, the ability of VA Health Connect nurses and providers to resolve the Veteran’s issue quickly, was described as one of the reasons why Veterans were satisfied with their experiences. A Veteran reflected on this issue, stating:

*I had never used telehealth before and as far as my first experience, it was phenomenal. . . I get the first call with the nurse, and she asks me the appropriate questions and then asks me if I would mind being seen by a provider via telehealth and I said absolutely not. I need to be seen by somebody, sooner rather than later. At this point it was like 6:30 in the morning local time for me. And she said, well, I got somebody available at 8:00 a.m. will that work? And I’m like, are you kidding me, that’s amazing. So yeah, it all worked smoother than I ever imagined that it could of*.

[Male Veteran, age 50 years, Northeast]

Several Veterans mentioned difficulties trying to schedule timely visits with their primary care providers (PCPs), particularly for acute issues, which require quick assessment and treatment; VA Health Connect offered them a way to resolve their health concerns quickly and alleviate some of the frustration they experience due to delays waiting for in-person care. To this point, a Veteran remarked:

*It was pleasant to be able to pick up the phone and get someone that could give me advice right away. You know, primary care sometimes takes a week or two to get an in-person visit*.

[Male Veteran, age 74 years, South]

Veterans lauded VA Health Connect services for providing care in a quick, timely manner, and also reported personal time savings due to having concerns resolved virtually rather than in person. Several Veterans mentioned having to travel long distances to get to the nearest VA or encountering traffic delays even when not geographically far, with some reporting driving up to 3 hours each way to get to their main VA hospital. Coupled with mobility issues or unreliable transportation options, VA Health Connect offered these Veterans an option that was convenient, accessible, and saved them time.

#### Clinical Advice and Recommendations

Veterans also reported high satisfaction with the clinical advice and recommendations provided by VA Health Connect nurses and providers. They described how staff accounted for their unique clinical circumstances and provided tailored advice, instilling their confidence in the information they received.

Elaborating on this issue, a Veteran explained:

*[It] was actually a really great experience for me . . . they were very informative. They gave me a lot of information. And when I explained my situation, they went through the exact steps they were going to do. They were going to put in the prescriptions for the antivirals, the antibiotics, and then we discussed the meds that I was currently on that I needed to stop taking during because there could be an interaction. And then they let me know that they would also be writing a notice, a letter, an official letter that I could—and emailing it directly to me with the information to give to the court to let them know why I was unable to do jury duty. And then about an hour or so later, he actually called me back again to let me know that he had finalized the prescription and asked me to check my email to make sure that I got it*.

[Female Veteran, age 39 years, South]

As this quote demonstrates, the provider went a step above and beyond by following up with the Veteran after the visit, which reaffirmed her confidence in the advice given.

One Veteran who went on to have an unsatisfactory subsequent health care experience outside the VA nevertheless thought that the original advice given by VA Health Connect providers and nurses was accurate and useful, commenting:

*I was going to say, however, unfortunately, what they recommended didn’t quite turn out the way that they had anticipated, but nonetheless, their service and recommendations were, I consider, spot on.*

[Male Veteran, age 55 years, South]

#### Organization of Services

The fourth reported reason for high Veteran satisfaction with VA Health Connect services was the overall organization of the services and the seamlessness with which care was delivered, including postvisit follow-up. Veterans were impressed with how quickly and without protracted, complicated additional steps they were able to resolve their health concerns, which they attributed to an optimally designed process.

One Veteran commented on this issue, stating:

*I know exactly who I need to talk to, but if I don’t, I just call the Health Connect nurse. First, I call the [VA location], and the operator comes on, and I just, I ask the operator, can I speak to the 24hour Health Connect nurse? Once I do that, the phone will ring, and it’ll pick up, and then they take care of me. I ain’t never had no problem with it*.

[Male Veteran, age 62 years, South]

Others mentioned specific aspects of their interactions that, for them, highlighted the efficiency and organization of the services. For example, a Veteran commented:

*Well, I appreciate this. They tell me either where I am on the queue or how many minutes it will take before I get answered. It’s very well done*.

[Male Veteran, age 85 years, West]

#### Positive Interactions With Providers and Nurses

Finally, Veterans reported highly satisfactory interactions with the nurses and providers whom they encountered through VA Health Connect. They described them as respectful, caring, professional, and knowledgeable.

Commenting specifically on his or her interactions with VA Health Connect nurses, one Veteran said:

*They’re very pleasant, very professional. They’re prompt. I mean, I really can’t speak highly enough about the triage nurses.*

[Male Veteran, age 62 years, South]

Another Veteran described how the triage nurse was able to keep him calm despite a stressful situation. He stated:

*The [nurse] . . . did a very good job staying focused … She handled me well. She kept me calm. And then got me exactly where I needed to get to*.

[Male Veteran, age 38 years, South]

Finally, other Veterans also noted how their interactions reinforce their perception that the VA both cares about and respects them as individuals. For instance, one said:

*The way they have treated me so far, I mean, there’s a lot of respect. None of them have been, oh, just acting like I’m just taking advantage of the process, if you understand what I’m trying to say there. They seem to care*.

[Male Veteran, age 55 years, South]

Another Veteran commented at length about VA Health Connect and telehealth services more generally:

*If my experience is the standard experience that a Veteran in need of care is going to get from telehealth, then I think the VA’s got a lot to be proud of. I mean you don’t rest on your laurels, there’s always room for improvement of course, but I just want to say thanks to the entire organization for taking such good care of Veterans . . . I was very pleased and very appreciative and happy to be an ambassador to extol virtues of my experience to others*.

[Male Veteran, age 50 years, Northeast]

### Areas for Increased Satisfaction and Improved Veteran Experiences

In general, Veterans reported very few challenges with VA Health Connect; however, several identified areas where their experiences could be improved or enhanced. One concern involved difficulties obtaining medications from community (nonVA) pharmacies after virtual visits, which created delays in accessing prescribed treatments. Another cited issue was the inability to bypass the nurse triage service and directly schedule an appointment with their PCP.

For example, one Veteran, while generally satisfied with his experience, expressed frustration about no longer being able to leave a voicemail for his nurse or PCP. Instead, he was routed through the triage line. Although he reported a satisfactory interaction with the tele-provider, he preferred the familiarity and continuity of an in-person visit with his regular PCP. He explained:

*I mean this is a small community here. And since that clinic is underutilized it’s—and I realize that can’t be everywhere, like you can’t do it in San Francisco or Los Angeles because the phones would just literally blow up probably. So, I can understand that they can’t. But I don’t know, it just seems they lost that personal [touch]. We have personal relationship with the folks down there*.

[Male Veteran, age 80 years, Northeast]

#### Veteran Awareness of On-Demand Clinical Care

While most Veterans were aware that VA Health Connect could assist with scheduling and pharmacy needs, they were largely unaware of the nurse triage and VCV services prior to calling the line. The majority of interviewees reported that they initially called VA Health Connect to schedule a visit with their PCPs to address an urgent health issue. Reflecting on how his interaction ultimately unfolded, one Veteran expressed satisfaction with the experience:

*I had never used it before. I’d never heard of it, [and] I don’t know how long it’s been in service, but it came across as if they had been actively doing this and had this down to a science. Because local hospitals have something similar, like [name of non-VA local healthcare system], for example. … And so, I thought, well, certainly the VA has got something that they could record and document that I called, and they can recommend what I should do based on VA services and what I can receive, and that’s exactly what they did. … I luckily stumbled upon it, and if you ask me right now to tell you to please tell me how I found it, I couldn’t tell you. I couldn’t tell you how to locate it again, unfortunately, to look them up, which is unfortunate because they were extremely helpful*.

[Male Veteran, age 55 years, South]

The last part of this Veteran’s comment also highlights another experience that some Veterans reported, namely, difficulty finding information about VA Health Connect and the specific number to contact for their needs.

This limited knowledge about the services available through the clinical contact centers suggests that greater Veteran outreach and education about the program is needed. When asked how the VA could help spread awareness of the services offered, Veterans provided several suggestions.

First, some suggested more advertising about VA Health Connect and the services available to Veterans. One Veteran stated:

*As a Veteran myself, I have never seen an advertisement for this. I didn’t know it existed . . . I didn’t know a triage center [exists], which really is an extremely valuable resource . . . That’s an invaluable tool to be able to have an RN or an LPN or even a med tech in some instances to be able to guide a patient and go, hey, you know what? You can go to this urgent care, and they can take care of your problem, but yet, man, you need to go to an ER*.

[Male Veteran, age 55 years, South]

Others suggested that PCPs inform patients about the services available, explaining to Veterans how it can be effectively used as a supplement to the care that they receive through primary care. A Veteran said:

*I would go to the doctors in the hospital, round them up and say, hey, listen, you guys are overburdened, why don’t you push people into the digital triage and that way we can offset it to a Call Center and do more digital triaging. See if you can get some buy-in from them and they’ll push it for you*.

[Male Veteran, age 38 years, South]

Others thought that local clinics and the VA at large could do a better job advertising the services, either through outgoing messages or pamphlets. One Veteran described searching on the VA’s website for information about the nurse triage line but had difficulty locating the correct number, suggesting that improvements to communication remain:

*I think clinics [need to make sure] they have the information on their website. Making sure that clinics have that information integrated into their outgoing messages. And then for some of our more mature patients, you know, maybe just good old fashion communicating with pamphlets so when a doc or a PA is there meeting with you in-person you can say, oh, by the way, if you ever need the VA’s assistance and it’s outside our hours of our operations, here’s something to keep on your nightstands for future reference*.

[Male Veteran, age 50 years, Northeast]

## Discussion

### Principal Findings

Veteran interviews revealed high satisfaction with VA Health Connect’s nurse triage and VCV services. Most notably, they appreciated the timeliness and convenience of being connected with a clinical provider, typically within 12 hours of their initial triage. This expedited access saved them time and potential costs associated with in-person urgent or emergency care visits. Veterans also described nurses and providers as knowledgeable and informative, which reinforced their trust in the care provided.

Despite overall positive experiences, participants also identified several limitations. A common concern involved Veterans who called VA Health Connect seeking to schedule an appointment with their PCP but were instead routed to the nurse triage line, leading to confusion or frustration. This mismatch points to an opportunity for better expectation-setting early in the interaction. Clearly explaining the types of services offered, and how they complement or substitute for other care options such as PCP visits or urgent and emergency care, could help align expectations and improve satisfaction for both patients and providers. Although not a theme among participants in this evaluation, some Veterans may also encounter barriers to virtual care due to limited digital literacy or physical and sensory challenges, which may make virtual care an untenable substitute for in-person care [12,13]. These well-documented challenges underscore the need for an array of options to be made available to patients.

Many Veterans were unaware of the nurse triage and VCV services prior to their initial use, suggesting more opportunities for the VA to advertise these services to Veterans. Participants also suggested several strategies to increase awareness, including traditional outreach methods such as pamphlets, website updates, and social media outreach. They also emphasized the role of PCPs in promoting and legitimizing these services. When PCPs frame virtual urgent care as a trusted extension of their own services, particularly for acute concerns that cannot be addressed promptly in person, Veterans may be more likely to accept and use these options.

More broadly, the findings underscore the importance of emphasizing choice in health care for Veterans. Recent policies such as the Choice Act, the Maintaining Internal Systems and Strengthening Integrated Outside Networks Act, and the priorities of current VA leadership reflect a commitment to empowering Veterans with options [14]. Yet, many Veterans still struggle to navigate the complexity of VA’s service offerings, often due to a lack of clear and timely information [15-17]. To address this, VA may need to use more creative and consistent communication strategies to keep Veterans informed about evolving services. This could include both traditional outreach and more consumer-oriented approaches, such as targeted marketing campaigns or app-based notifications—methods commonly used in the private sector [18]. Where feasible, technology may serve as a powerful tool for spreading awareness, especially among digitally engaged patients.

In addition to improving awareness and communication, it is important to recognize the broader value these services offer to Veterans. While virtual triage and urgent care programs may not generate substantial direct cost savings for the health care system, they clearly deliver value to patients. Benefits include improved access to timely care, reduced travel and transportation burdens, and high levels of patient satisfaction. These patient-centered outcomes are critical measures of success in their own right [19].

### Limitations

Our sampling strategy, while effective for facilitating recruitment, may have introduced selection bias by overrepresenting Veterans who had positive experiences with VA Health Connect. Actively seeking out Veterans with less favorable experiences in future research could uncover additional opportunities for program improvement. In addition, because our study focused exclusively on Veterans within the VA system, the findings may not be generalizable to nonVA settings or populations. Additional research on Veterans’ experiences with using VA Health Connect could also explore the role that the acute condition for which Veterans are calling plays in their overall satisfaction and experience with VA Health Connect, as some conditions may be better suited for virtual care.

### Conclusions

Veterans who recently used VA Health Connect’s nurse triage and VCV services reported high levels of satisfaction and a strong willingness to use these services again. Key strengths included timely access, clear communication, and professional, respectful interactions with nurses and providers. However, the findings also highlighted opportunities for improvement, particularly in communication and outreach. Many participants were unaware of the services prior to using them, underscoring the need for broader promotion. Setting expectations early, particularly around what VA Health Connect can and cannot provide, may further enhance the experience for both Veterans and providers. As integrated virtual triage and follow-up care become more widespread, a key challenge will be integrating these services more fully into routine care so they are consistently recognized, trusted, and used as part of patients’ broader care experience.

## Supplementary material

10.2196/80656 Multimedia Appendix 1 Interview guide.

## Abbreviations

PCP: primary care provider
RN: registered nurse
VA: Veterans Affairs
VCV: virtual clinical visit
