# Supplementary material for: Veteran Experiences and Satisfaction With Veterans Affairs Call Centers’ Tele-Triage and Virtual Urgent Care Appointments: Qualitative Evaluation
Source: J Med Internet Res. 2025 Nov 11;27:e80656. doi: 10.2196/80656 (PMC12604740; doi:10.2196/80656)
Supplement: Multimedia Appendix 1 [file jmir-v27-e80656-s001.docx]

**Questions:**

1. You recently contacted the call center on [date]. Can you describe for me in your own words why you made this call? How did you hear about this service? What were your overall thoughts about this experience?
2. Describe for me what happened during your call. Was it transferred to different lines? If so, what was that experience like? Did it proceed smoothly, or were there some challenges? (Probes: selecting the right option from the menu, being transferred to the correct location, etc.)
3. Did you have any technical issues during your call? If so, what were they and did you come up with any ways to deal with those issues?
4. Was your issue resolved to your satisfaction? Why or why not? How could the call have been improved?
5. Describe your impressions of the persons who you interacted with on the other end of the call (MSAs, nurses, providers, etc.). Were they helpful/friendly/other descriptors? How or how not?
6. [For **Clinical Triage**], what was your interaction like with the nurse?
   1. What did he/she recommend you do? Did you follow his/her recommendation (regarding both time and destination)? Why or why not?
   2. How could your interaction with the nurse have been better, if at all?
   3. What care did you use after your call?
      1. ED (VA), ED (Community), PCP used the Nurse triage/CCC program again, None
7. [For **Virtual Services**],
   1. In what way did you interact with the provider - over the phone or via tele-video? How was the visit type determined?
   2. Do you prefer one method over the others? If so, which one and why?
   3. Did you feel satisfied with the assessment the provider gave you? Was it thorough? Why or why not? Do you feel like they were able to adequately assess your situation? Why or why not?
   4. Did you feel satisfied with your visit? Why or why not? How could it have been improved, if at all?
   5. Did you think your visit was comparable to in person care? Why or why not?
   6. Would you have come in-person to the emergency department if virtual care was not available? If virtual care was not available, would you have gone to: VA ED, Community ED, Community urgent care, VA primary care, Non-VA primary care, No care
   7. Would you like to receive care like this again? Why or why not?
8. What do you like most/like least about the way your call was managed?
9. Was there any follow up after your call? Did you expect there to be? What would you have liked to have happened after your call, if anything?
10. How frequently do you use the call center? Has this changed in recent years/months? If so, why? Would you like to use the call center more frequently? Why or why not?
11. Would your recent experience with the call center make you more or less likely to use the VA for future services? Please explain why.
12. How could we improve your experiences with VA call centers?
